# Predictors of peritoneal metastasis of gastric origin

**DOI:** 10.1186/s43046-022-00155-y

**Published:** 2022-12-19

**Authors:** Mohamed Atef ElKordy, Rady Mansour Soliman, Mahitab Ibrahim ElTohamy, Dalia Negm Eldin Mohamed, Ahmed Morsi Mustafa

**Affiliations:** 1 Department of Surgical Oncology, NCI, Cairo University, Cairo, Egypt; 2 Department of Pathology, NCI, Cairo University, Cairo, Egypt; 3 Department of Statistics, NCI, Cairo University, Cairo, Egypt

## Abstract

**Background:**

Gastric adenocarcinoma is one of the most aggressive forms of cancer. Despite marked advancements in radiological techniques, peritoneal deposits are still only discovered during laparotomies in a significant number of cases. The role of surgery in the management of metastatic gastric cancer is very limited, reducing the value of conducting laparotomies. In addition, conducting laparoscopies for the purposes of properly staging every case of gastric cancer is difficult, especially in healthcare systems with limited resources. It is thus crucial to investigate all possible predictors of peritoneal metastasis of gastric cancer, with the aim of reserving the use of laparoscopies to cases known to have high incidences of peritoneal metastasis despite negative radiological results.

**Patients and methods:**

This is a case control study that included all cases of gastric adenocarcinoma that had presented to the National Cancer Institute–Cairo University between January 2018 and December 2019. The ‘cases’ group encompassed all gastric adenocarcinoma patients who were found to have peritoneal metastasis, whilst the ‘control’ group included those patients who were apparently metastasis-free. Comparisons were made between the two groups in terms of demographics, tumor characteristics, and results of laboratory tumor marker investigations.

**Results:**

Patients with peritoneal metastasis were statistically significantly younger than those who had no apparent metastasis (mean ± SD 51.4 ± 12.5 and 56.2 ± 12.6 respectively; *P* = 0.020). Significant associations were found between a finding of peritoneal metastasis and (i) a middle tumor site (*P* = 0.002); (ii) tumor thickening morphology (*P* < 0.001); (iii) undifferentiated histopathology (*P* = 0.040); (iv) tumor grade III (*P* < 0.001); (v) lower lymphocyte counts of < 1.9/ml (*P* = 0.030); and (vi) high levels of CA 19-9 of > 37 units/ml (*P* = 0.032).

**Conclusion:**

Tumor pathological criteria, including tumor site, degree of differentiation, shape, and grading, as well as laboratory findings of low lymphocytic counts and high levels of CA 19-9 appear to be reliable predictors of the presence of peritoneal metastasis from a gastric adenocarcinoma.

## Background

Globally, gastric cancer is one of the most aggressive forms of cancer. In 2020, 1.09 million new cases of gastric cancer were reported worldwide, leading to approximately 769,000 deaths in the same year. As such, adenocarcinoma of the stomach is currently the fourth most common cause of death due to a malignancy worldwide [1].

According to the SEER registry, significantly wide differences in survival rates of patients with distantly metastatic gastric cancer compared with those with only localized or regional disease were recently reported. The 5-year survival of gastric cancer currently stands at 69.9, 32.4, and 5.5% in localized, regional, and distant metastatic cancer respectively [2]. These figures highlight the importance of developing new strategies for the early detection and prediction of advanced disease.

In terms of improving survival, the role of surgery in metastatic gastric cancer is presently very limited. Imaging studies are of limited sensitivity in the detection of peritoneal metastasis [3] and preforming laparoscopies for all cases to detect metastases would entail high costs that some healthcare systems cannot afford. It thus becomes crucial to find reliable predictors of peritoneal metastasis that would help cut down on the number of laparotomies performed. Such predictors may also become part of a refined approach to the detection of metastases in cases of gastric adenocarcinoma, reserving laparoscopies to cases where metastases are highly suspected.

## Aim of work

To identify predictors (risk factors) of peritoneal metastasis resulting from gastric adenocarcinoma.

## Patients and methods

This is a case-control study which included all cases with gastric adenocarcinoma who had presented to the National Cancer Institute (NCI)–Cairo University and were managed there between January 2018 and December 2019. The ‘cases’ group included patients who had either presented with peritoneal deposits or developed them during follow-up. The ‘control’ group was comprised of patients who had not presented with peritoneal deposits nor developed them during follow up. Peritoneal metastasis were diagnosed during presentation using the standard protocol in NCI for gastric cancer; as all cases underwent CT abdomen and pelvis with IV and oral contrast OR PET-CT, if their results were negative for peritoneal or distant metastasis, the patients were admitted for diagnostic laparoscopy before surgery or starting neoadjuvant therapy. While the other cases who developed peritoneal metastasis after surgery were diagnosed using the follow up protocol as follow:

During the 1st 2 years: history and examination were done every 3 months and CT abdomen and pelvis with IV and oral contrast every 6 months

During the following 3 years: history and examination every 6 months and CT Abdomen and pelvis with IV and oral contrast annually.

Guided biopsy were taken from lesions only if the imaging results were unsure.

Inclusion criteria included a minimum of 1 year of follow-up.

The two groups were compared in terms: (i) demographics and clinical findings, including age, gender and presenting symptom(s); (ii) tumor characteristics, including the location of the primary tumor (upper, middle, or lower stomach), tumor morphology, classified as polypoid type, ulcerated type, or thickening (infiltrative type), T stage (depth of tumor invasion) and N stage (nodal status), histopathological type, defined as either an adenocarcinoma (NOS), mucinous carcinoma, Signet ring carcinoma, or undifferentiated tumor, and histological grade, labeled as G1, 2, or 3; (iii) laboratory findings, including neutrophil and lymphocyte counts, N/L ratio, and albumin level; and (iv) levels of the tumor markers CEA and CA19-9.

## Statistical method

Data were analyzed using computer program IBM SPSS (Statistical Package for the Social Science; IBM Corp, Armonk, NY, USA) release 21 for Microsoft Windows. Numerical data were tested for the normal assumption using Kolmogorov Smirnov test. Normally distributed variables were described as mean ± standard deviation (±SD), median and range, categorical variables were described as frequencies (number of cases) and percentages. All numerical data were normally distributed except CEA and CA19-9. All variables were further divided into two groups considering their median as the cut-off value. For comparing prognostic factors with the presence or absence of peritoneal metastasis, chi-square (*χ*^2^) test was performed. Exact test was used instead when the expected frequency is less than 5. Univariate followed by multivariate binary logistic regression was performed for all statistically significant variables on the univariate analysis to identify independent factors affecting the occurrence of peritoneal metastasis. *P* values less than 0.05 was considered statistically significant, and all tests were two tailed.

## Results

Between January 2018 and December 2019, 203 patients were diagnosed with gastric adenocarcinoma at the National Cancer Institute. Peritoneal metastasis of gastric origin was detected in 59 (29.1%) patients, whilst 144 (70.9%) were apparently peritoneal-metastasis free.

The mean age of patients at presentation stood at 55 years of age. There was a small majority of male patients (55%) compared with female patients (45%). Vomiting was the main presenting symptom (38.4%).

Statistical analysis revealed a number of associations between peritoneal metastasis and a number of the aforementioned patient and tumor characteristics and laboratory findings (see Table 1). Patients with peritoneal metastasis were found to have been statistically significantly younger than those with no apparent metastasis (mean ± SD 51.4 ± 12.5 and 56.2 ± 12.6, respectively, *P* = 0.020). Significant associations were found between a finding of peritoneal metastasis and (i) a middle tumor site (*P* = 0.002); (ii) tumor thickening morphology (*P* < 0.001); (iii) undifferentiated histopathology (*P* = 0.040); (iv) tumor grade III (*P* < 0.001); (v) lower lymphocyte counts of < 1.9/ml (*P* = 0.030); and (vi) high levels of CA 19-9 of > 37 units/ml (*P* = 0.032).

**Table 1:** Association between different demographic, pathological and laboratory markers with presence of peritoneal metastases

Prognostic factor	Peritoneal metastasis				***P*** value
	No		Yes		
	***N***	%	***N***	%	
Age (year)	56.2 ± 12.6		51.4 ± 12.5		0.02
Gender					
Male	85	59.0	27	45.8	
Female	59	41.0	32	54.2	0.084
Site of the tumor^a^					
Distal	52	36.1	9	15.3	
Middle	45	31.3	33	55.9	
Proximal	47	32.6	17	28.8	0.002
Morphology					
Polypoidal mass	110	76.4	38	64.4	
Thickening	7	4.9	14	23.7	
Ulcer	27	18.8	7	11.9	< 0.001
T stage^b^					
T2	8	11.9	1	5.0	
T3	25	37.3	6	30.0	
T4	31	46.3	13	65.0	0.523
N stage ^b^					
N0	24	35.8	2	10.0	
N1	14	20.9	6	30.0	
N2	18	26.9	5	25.0	
N3	11	16.4	7	35.0	0.074
Histological type					
Adenocarcinoma NOS	98	68.1	29	49.2	
Mucinous adenocarcinoma	5	3.5	3	5.1	
Signet ring adenocarcinoma	38	26.4	23	39.0	
Undifferentiated carcinoma	3	2.1	4	6.8	0.040
Grade					
Grade I	4	2.8	1	1.7	
Grade II	95	66.0	20	33.9	
Grade III	45	31.3	38	64.4	< 0.001
Neutrophils					
<= 4.17	56	41.2	20	34.5	
> 4.17	80	58.8	38	65.5	0.382
Lymphocytes					
<= 1.9	64	47.1	37	63.8	
> 1.9	72	52.9	21	36.2	0.033
NLR					
<= 2.6	72	53.7	26	47.3	
> 2.6	62	46.3	29	52.7	0.420
Albumin					
<= 3.5	68	59.6	27	56.3	
> 3.5	46	40.4	21	43.8	0.688
CEA					
<= 5	54	69.2	23	57.5	
> 5	24	30.8	17	42.5	0.205
CA19-9					
<= 37	53	71.6	20	51.3	
> 37	21	28.4	19	48.7	0.032

^a^The upper part of stomach includes the gastro-esophageal junction and cardia; the middle part includes the body of stomach, while the distal part includes the pyloric antrum and pyloric canal

^b^Not all cases had undergone endoscopic ultrasounds and T and N staging were thus missing from a number of patient medical records

Table 2 shows the results of univariate logistic regression analysis of all statistically significant variables conducted using the chi-square test. Multivariate logistic regression analysis was performed (Table 3) to identify independent predictors of peritoneal metastasis. The following tumor characteristics were found to be independent predictors of peritoneal metastasis: (i) diffuse thickening morphology (OR = 4.832, 95% CI = 1.671–13.972, *p* = .0004); (ii) histopathological grade III (OR = 3.944 95% CI = 1.989−7.817, *p* < 0.001); and (iii) middle part tumors (OR = 2.8510, 95% CI = 1.2587–6.4576, *p* = 0.012).

**Table 2:** Univariate logistic regression analysis

	***B***	***P*** value	OR	95% C.I. for OR	
				Lower	Upper
Age	− 0.029	0.017	0.971	0.948	0.995
Thickening morphology	1.806	< 0.001	6.089	2.313	16.026
Signet ring and undifferentiated carcinoma	0.751	0.019	2.12	1.132	3.969
Grade III	1.382	< 0.001	3.981	2.101	7.541
Lymphocyte <= 1.9	0.684	0.034	1.982	1.053	3.731
Site: lower(reference)		0.002			
Middle	1.444	0.001	4.237	1.833	9.796
Upper	0.737	0.108	2.09	0.85	5.136
CA19-9> 37	0.874	0.033	2.398	1.071	5.368

**Table 3:** Multivariate analysis for independent factors that predict peritoneal metastasis

	***B***	***P*** value	OR	95% C.I.	
				Lower	Upper
Morphology (thickening)	1.575	.004	4.832	1.671	13.972
Grade (III)	1.372	< 0.001	3.944	1.989	7.817
Distal (reference)		.014			
Middle	1.335	.003	3.799	1.555	9.277
Proximal	.877	.071	2.405	.927	6.239
Constant	− 2.586	< 0.001	.075		

## Discussion

Adenocarcinoma of stomach has a high capability of sending peritoneal deposits. The benefit of surgery in the term of cure or improving survival in these cases was very limited. Unfortunately, many cases were discovered to have peritoneal deposits only during surgery in spite of negative imaging findings regarding peritoneal deposits, making theses surgeries invaluable and conducting laparoscopy for all patients with gastric cancer is costly and maybe unavailable in all centers especially in healthcare system of limited resources and hence the importance of the conducting study which aimed to identify the factors which make the biology and the stage of gastric cancer have higher incidence of sending peritoneal deposits and so limiting the laparoscopic exploration for only this cases. An important finding of the current study was the association of a tumor in middle part (body) of stomach with higher incidence of peritoneal deposits in contrast to tumors originating from the cardia or pylorus. This may be attributable to the low incidence of obstructive symptoms of gastric cancer associated with body tumors compared with tumors originating from other parts of the stomach, delaying patient presentation to late stages of the disease. Irene Thomasson et al. reported that, in contrast to tumors located in only one region of the stomach, tumors which occupied more than one anatomical part of stomach were associated with higher incidences of peritoneal metastasis [4]. Diffuse infiltration morphology which usually present in linitis plastica and aggressive pathology like undifferentiated carcinoma were associated with higher incidence of peritoneal carcinomatosis. This is concomitant with C Honoré et al. findings which showed the same results [5, 6]. In concordance with Florian Seyfried et al. findings, high grade tumor (G3) had higher incidence of peritoneal metastasis (*P* value = 0.018).

Rosenberg R., et al. explained that peritoneal carcinomatosis develops through the penetration of tumor cells into the serosa, followed by their shedding into the peritoneal cavity [7]. In addition, the deeper the penetration of tumor cells into the layers of stomach, the more these tumor cells have access to the lymphatic system, leading to higher incidences of peritoneal metastasis. The conducting study showed higher trend of the percent of the cases with peritoneal deposits in advanced T stage (T3, T4) in comparison to T2 but statistically insignificant. In Literature, Michael D’ Angelica et al. reported peritoneal recurrence in 37% of the cases of completely resected T3 and T4 gastric carcinomas versus a recurrence rate of only 13% of cases diagnosed with T0, T1, and T2 tumors [8].

Detailed interactions between tumor and inflammatory cells have not, to date, been fully delineated. Neutrophils could possibly have a role in the promotion of tumor formation through the production of vascular endothelial growth factor and matrix metalloproteinase-9 [9, 10]. In addition, the neutrophils are believed to have inhibitory effects on natural killer cells and lymphocytes, possibly promoting the progression of tumors [11, 12]. It has also been proposed that activated platelets may have a role in the progression of tumors through the secretion of growth factors [13]. Conversely, the role of the adaptive immune system, especially lymphocytes, in the eradication of cancer cells has been extensively studied and further affirmed after the finding of increase incidence of cancer in patients receiving immunosuppressants after organ transplantation [14]. Robert Schreiber reported an increased incidence of sarcomas in mice which lacked an adaptive immune system [15]. In the present study, lymphocytic count below or equal 1.9 × 10^3^ per microliter was a found to be risk factor for peritoneal spread of a gastric carcinoma.

A large number of studies have attempted to create a comprehensive model of the role of immune factors in cancer development and spread in order to better predict prognosis and recurrence. Such immune factors have included a number of ratios, including neutrophils/lymphocytes, platelets/lymphocytes, and monocytes/lymphocytes [16, 17]. Nakayama et al. reported that a neutrophil/lymphocytic ratio > 2.37 was an independent predictor of peritoneal metastasis of gastric origin [18]. Inoue, et al. reported higher peritoneal recurrence rate and lower overall survival after gastrectomy in cases with a high preoperative systemic immune inflammation index of S II (with a cut-off 395, *P* value = 0.028). The index is computed as follows: platelet count × neutrophil count/lymphocyte count [19].

The conducted study highlighted the impact of different factors on the presence or absence of peritoneal metastasis of gastric origin but it has some limitations regarding the retrospective design and some missing data like exactly T and N stage of the tumors for some patients. But its results are valuable as it showed that the site ,morphology and grading of tumor as well as low lymphocytic count and higher CA 19-9 were predictors for peritoneal metastasis from gastric cancer. Further studies needed to be conducted comparing laparoscopic exploration findings regarding presence or absence of peritoneal deposits with the predicting factors and discuss the interaction between them aiming to exactly identify the exact patients who will benefit from laparoscopy.

## Conclusions

A number of pathological characteristics of gastric tumors including site, degree of differentiation, shape and grading, as well as a low lymphocytic count and high levels of CA 19-9 may be predictors of peritoneal metastasis from gastric adenocarcinoma.

## Data Availability

The datasets used and analyzed during the current study are available from the corresponding author on reasonable request.

## Abbreviations

SEER: Surveillance, Epidemiology, and End Results Program
CI: Confidence interval
OR: Odds ratio
CEA: Carcino-embryonic antigen
CA19-9: Carbohydrate antigen
